# AA5052-PVC-AA5052 (Al-PVC-Al) Sandwich Sheets Forming Analysis through In-Plane Plane Stretching Tests

**DOI:** 10.1155/2024/5117746

**Published:** 2024-03-08

**Authors:** P. Praveen Kumar Reddy, Chinmaya Prasad Padhy, P. Janaki Ramulu

**Affiliations:** ^1^Department of Mechanical Engineering, School of Technology, GITAM Deemed to Be University, Hyderabad, India; ^2^Department of Mechanical Engineering, Adama Science and Technology University, Adama 1888, Ethiopia

## Abstract

Sheet metal forming is one of the key processes for the automotive sector to be considered. Sheet metal formability is being tested as received, joining them with different welding/joining processes (i.e., tailored blanks) and making them as sandwich forms to reduce the total weight of the body. These sandwich formations of sheets are an advanced method by incorporating PVC/polymer sheets in between metal sheets with a suitable binder. The present work has investigated the formability of AA5052-PVC-AA5052 (Al-PVC-Al) sandwich sheets by considering the sheet rolling direction as a parameter. The mechanical properties of base metal and sandwich sheets were evaluated by conducting the uniaxial tensile tests. For forming behaviour of Al-PVC-Al sandwich sheets, in-plane plane stretching tests were performed on the universal tensile testing machine. From the results, it has been observed that 0-degree and 90-degree rolling direction of AA5052 sheets provided almost similar forming behaviour where the 45-degree rolling direction showed less formability. The limit strains (by which the forming limit curve has been developed and the safe and failure zones are separated) are 0.043, 0.038, and 0.043 of 0°, 45°, and 90°, respectively. Considering 0°-P-90°, 90°-P-90, 0°-P-45°, 0°-P-90°, and 45°-P-45° sandwich sheets with their corresponding limit strains of 0.060, 0.058,0.057, 0.052, and 0.050, a better formability is seen in 0°-P-90° sandwich, followed by 90°-P-90, 0°-P-45°, 0°-P-90°, and 45°-P-45°. The improvement in the formability is calculated as 28.33%, 25.86%, and 24.0% in comparison with the base metal in 0-degree, 90-degree, and 45-degree rolling directions and 0°-P-90°, 90°-P-90, and 45°-P-45° sandwich sheets.

## 1. Introduction

Forming behaviour analysis of any sheet metal leads to the proper application in an industry sector. Identifying the forming limit strains and constructing forming limit curve/diagram (FLC/D) are major concerns for sheet metals. The number of ways to construct FLCs and their significance has been represented by many researchers; a few of them are explained inTable 1.

A different concept has been introduced on double-walled porous functionally graded magneto-electro-elastic sandwich plates by Saffari et al. [24]. In this study, we investigated the effect of uniform and nonuniform temperature distributions on sound transmission loss on the sandwich plates with subsonic external flow.

Fabrication and the formability of the various sandwich sheets made of Al alloy-based, steel-based, Cu-based, and Al and steel combination along with polymers have been observed. From the above-stated forming studies, it has been noted that deep drawing, stretching, and in-plane plane stretching tests are used for formability analysis by considering sandwich thickness, test parameters, machine capabilities, metal-polymer binder capabilities, sheet rolling directions, and sheet dimensions, etc., as main parameters. Moreover, the impact of sheet rolling direction in detail has not been reported yet. The present work aimed to establish the FLCs of Al-polymer-Al sandwich sheets with respect to three rolling directions (0°, 45°, and 90°) and their combined effects (0°–0°, 0°–45°, 45°–45°, 0°–90°, and 90°–90°).

## 2. Experimental Methodology

### 2.1. Base Materials and Specimen Preparation

**Figure 1:**
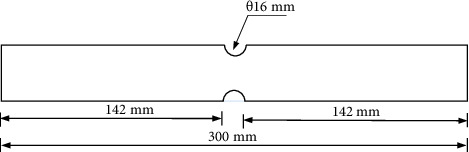
Schematic representation of an in-plane plane-strain tensile test specimen.

**Figure 2:**
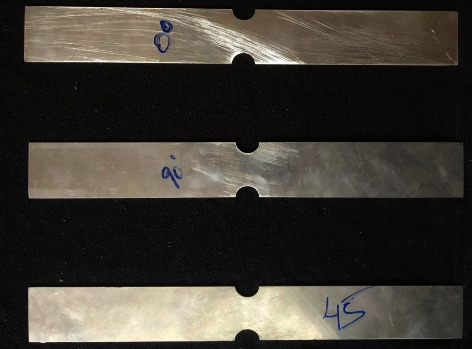
In-plane plane stretching specimens of the AA5052 alloy sheet in 0°, 45°, and 90° rolling directions.

The base metal AA5052 alloy sheet of 1 mm thickness and ultraclear PVC sheet of thickness 0.5 mm were selected as sandwich sheets. The AA5052 sheet was cut in 0°, 45°, and 90° rolling directions with the dimensions as shown in Figure 1. Based on these dimensions, AA5052 sheet specimens were cut using a CNC milling machine in three rolling directions as shown in Figure 2. The PVC sheet was also cut with the same dimensions. The surfaces of the AA5052 sheets were cleaned before the binder was applied. Equal quantities (by volume) of resin and hardener of Araldite Standard were taken and mixed until it forms a uniform colour. A thin coat of the mixture was applied on both the surfaces of AA5052 specimen sheets and PVC sheets inserted in between them. The pressure was applied using *c*-clamps such that excess resin came out. For proper adhesive bond formation, Al-P-Al sandwich sheets were kept up to 6–8 hours under uniform clamping conditions. Afterwards, *c*-clamps were removed, and sandwich sheets were kept idle for 24 hours; later, all the sandwich sheets were cleaned and taken for further tests.

**Figure 3:**
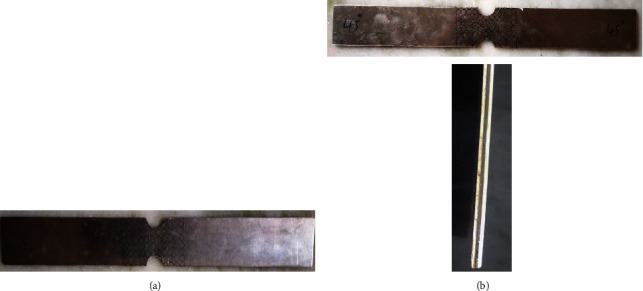
In-plane plane stretching specimen of the base metal sheet and Al-PVC-Al sandwich sheet. (a) AA5052 alloy sheet. (b) AA5052-PVC-AA5052 sandwich sheet of 45° rolling direction (top) and thickness direction of sandwich (bottom).

Sandwich sheets were made with a combination of different rolling directions of AA5052 such as 0°-P-0°, 45°-P-45°, 90°-P-90°, 0°-P-45°, and 0°-P-90° (P refers PVC). On base metal and sandwich sheets, a circular (5 mm diameter) grid pattern was stamped near the grooved (effective) area (Figure 3).

The obtained limit strains were used for developing the FLC for each case. For the initial limit strain of FLC, FLC_0_ was calculated by Keeler and Brazier [25], and equation (1) resulted in a relationship between the FLC_0_ and sheet thickness (*t* in mm) and the strain hardening exponent (*n*).

(1) $$\begin{array}{c} {\text{FLC}}_{0}=\left(0.233+0.143t\right)\times \left(\frac{n}{0.21}\right). \end{array}$$

The details of forming a limit diagram with various zones are described in [4]. The calculated major strains and minor strains, i.e., limit strains, from the various formability tests were plotted to identify the forming limit curve (FLC).

### 2.2. Tensile Testing of Base Metal Sheet and Sandwich Sheets

Uniaxial tensile tests were conducted using the MCS computerised universal testing machine of 1-ton capacity. The specimens of base metal sheet AA5052 and AA5052-PVC-AA5052 were made based on the width constraint method. All the tests had been conducted with a strain rate of 1 mm/min, and reputation was also maintained for each sample. From the base sheet of AA5052, samples were cut in three different rolling directions, namely, 0°, 45°, and 90°, whereas sandwich sheets were made with a combination of different rolling directions of AA5052, such as 0°-P-0°, 45°-P-45°, 90°-P-90°, 0°-P-45°, and 0°-P-90° (P refers PVC).

## 3. Results and Discussion

As per the previous section explanation, as received based on AA5052 alloy sheet of 1 mm thickness along with 0.5 mm thickness, PVC sandwich sheets have been fabricated and tested for mechanical properties and in-plane plane stretching tests.

### 3.1. Mechanical Properties of AA5052 Sheet and Sandwich Sheets

The strain hardening exponents of the AA5052 alloy sheet and Al-PVC-Al sandwich sheets were evaluated using uniaxial tensile test. The obtained results of yield strength and strain hardening exponent are tabulated in Tables 2 and 3.

From the results of tensile tests of the base sheet and sandwich sheets, 0° and 90° showed similar trends in the base sheet, whereas the 90°-P-90° sandwich sheet has more strain hardening value compared to other sandwich sheets.

### 3.2. In-Plane Plane Stretching Tests

**Figure 4:**
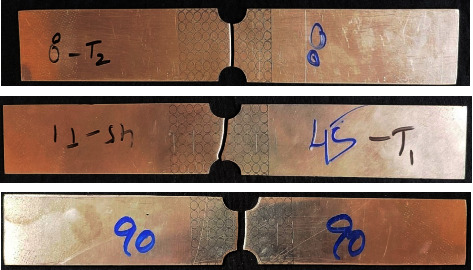
AA5052 alloy sheet in-plane stretching tested specimens.

**Figure 5:**
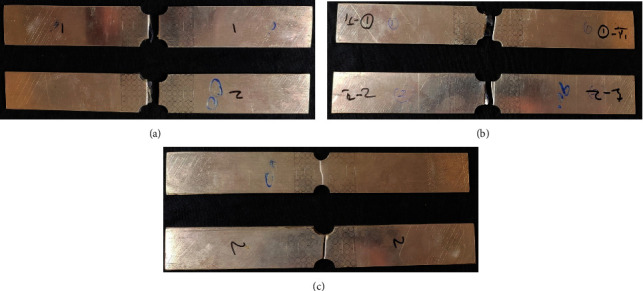
In-plane stretching tested specimens of Al-P-Al sandwich sheets. (a) 0°-P-0° sandwich sheet. (b) 0°-P-90° sandwich sheet. (c) 0°-P-45° sandwich sheet.

In-plane plane stretching tests were performed on a universal testing machine under a constant strain rate of 1 mm/min which made sure that none of the samples failed in the gripping zone. The test specimens were marked with circular grids of 5 mm diameter in the effective zone. The failure phenomenon has been observed similarly in the rolling directions. For illustration, AA5052 alloy sheet specimens of 0°, 45°, and 90° failed in in-plane stretching test are shown in Figure 4, respectively. In a similar manner, the failure phenomenon is also seen in Al-P-Al sandwich sheets (Figure 5).

### 3.3. Limit Strain Calculations and Developing Forming Limit Diagram

From the tested AA5052 alloy sheet, the deformed zone was identified and the major and minor strains were measured using equation (1) as mentioned in Section 2. From the obtained major and minor strains, FLDs were developed for base AA5052 alloy sheets and Al-P-Al sandwich sheets corresponding to their rolling directions. The initial limit strain for forming limit curve (FLC_0_) was evaluated using equation (1).

#### 3.3.1. FLDs of AA5052 Alloy Sheets

**Figure 6:**
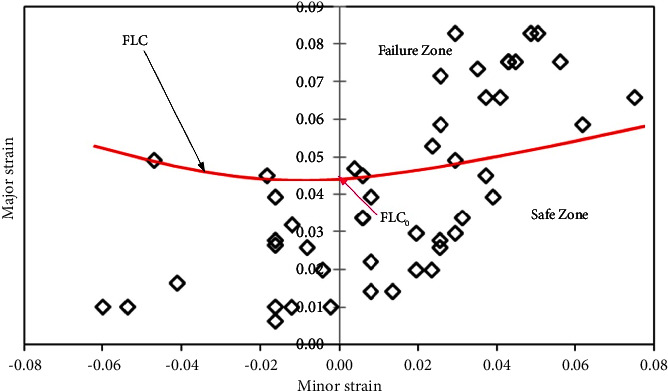
Forming limit diagram of the 0-degree rolling direction of the AA5052 alloy sheet.

**Figure 7:**
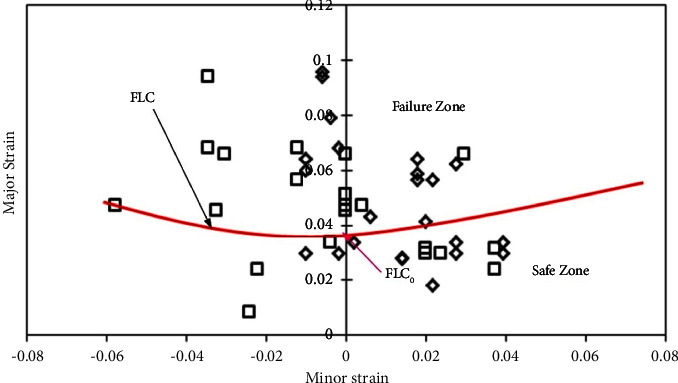
Forming limit diagram of the 45-degree rolling direction of the AA5052 alloy sheet.

**Figure 8:**
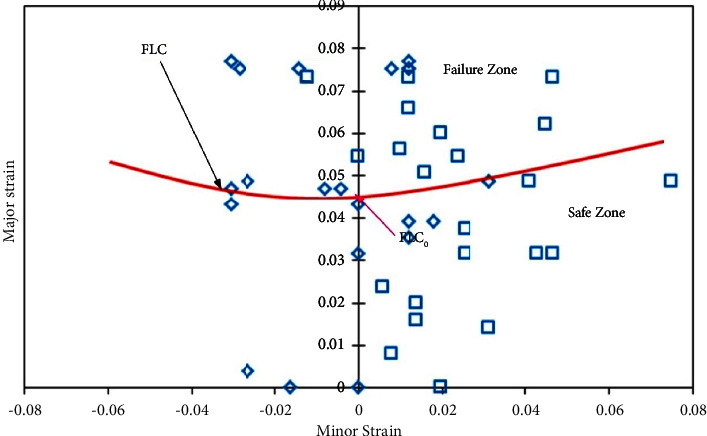
Forming limit diagram of the 90-degree rolling direction of the AA5052 alloy sheet.

Figures 6–8 show the FLDs of the AA5052 alloy in-plane plane stretching testing specimen with sheet rolling direction as a parameter.  Figure 6 depicts the FLD of the 0-degree rolling direction sheet with the division of safe limit strains and failure zone. Based on equation (1), FLC_0_ is calculated by which FLC has been drawn.  Table 4 shows the limit strain values which are evaluated by using equation (1) for base metals. For this case, 0° and 90° base sheets show similar FL strains.

In a similar manner, for sheet 45- and 90-degree rolling direction sheets, FLDs have been developed (Figures 7 and 8) with the same approach. It has been observed that FLCs of 0- and 90-degree AA5052 sheet are the same, which have better formability compared to the 45-degree sheet. This difference is accounted for by variation in the strain hardening values (Table 2).

#### 3.3.2. FLDs of AA5052-PVC-AA5052 (Al-P-Al) Sandwich Sheets

The sandwich sheets have been fabricated with *0-P-0*, *0-P-45*, *45-P-45*, *0-P-90*, and *90-P-90* sheet rolling direction combinations to see the forming behaviour. Circular grid sampling was carried out on the sandwich sheets for limit strain calculations. During the test, none of the sheets failed in the grip zone. All sheets failed as per the expectation like first the metal layer and then the polymer. This is being noted in all the sheets.

The FLDs have been developed for all cases such as 0*-P-0*, *0-P-45*, *0-P-90*, *45-P-45*, and *90-P-90* sandwich sheet rolling direction combinations. Limit strain calculation has been performed based on the necking phenomenon which indicates the limit strains near to necking or failure of the sheet. For FLC_0_ identification, equation (1) does not show the suitability for limit strain evaluations. For this, necking /failure zone strains have been identified and separated from the safe zone and failure zone with manual FLC.  Table 5 shows the limit strain values of 0*-P-0, 0-P-45*, *0-P-90*, *45-P-45*, and *90-P-90* sandwich sheets by which safe and failure zones are separated. From the limit strain values (Table 5), the maximum limit strain has been noted for the *0-P-0* sandwich sheet, followed by *90-P-90*, *0-P-45*, *0-P-90*, and *45-P-45* sandwich sheets. From these values, better formability is seen for the *0-P-0* sandwich sheet with a slight variation to the *90-P-90* sandwich sheet.

**Figure 9:**
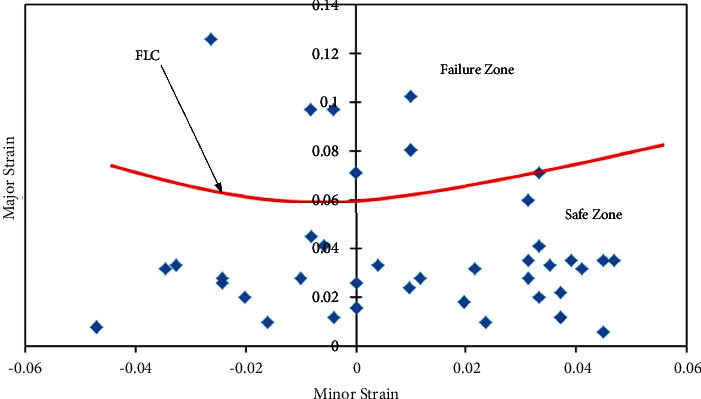
FLD of the *0-P-0* sandwich sheet.

**Figure 10:**
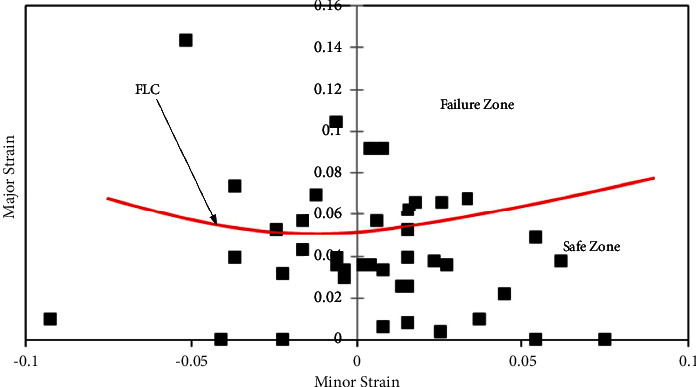
FLD of the *0-P-45* sandwich sheet.

**Figure 11:**
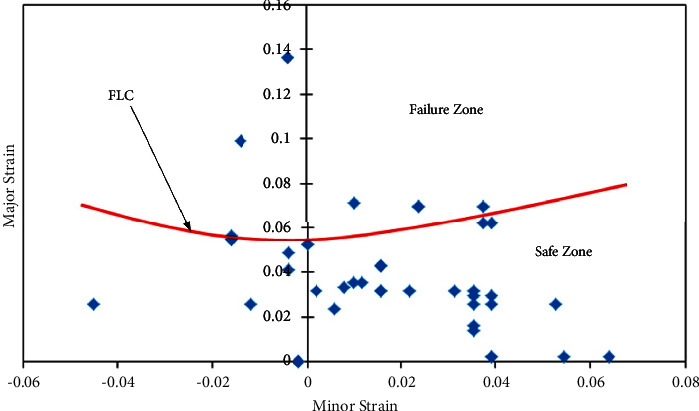
FLD of the *0-P-90* sandwich sheet.

**Figure 12:**
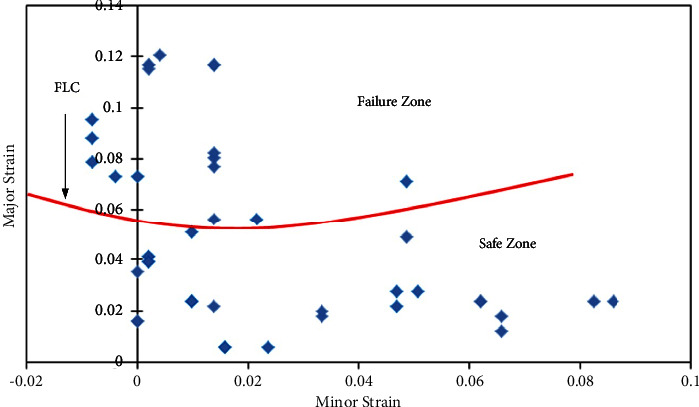
FLD of the *45-P-45* sandwich sheet.

**Figure 13:**
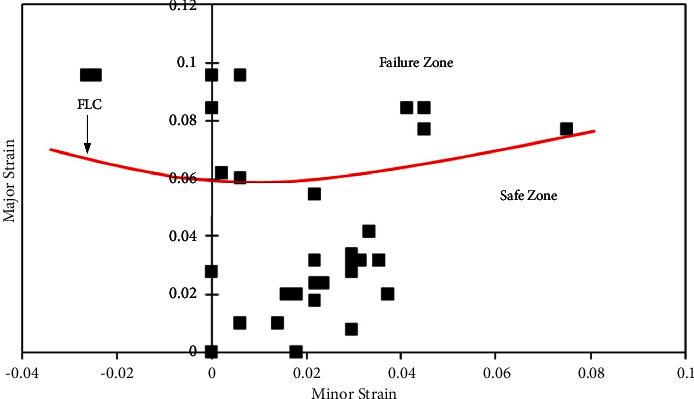
FLD of the *90-P-90* sandwich sheet.

Figures 9–13 show FLDs of *0-P-0*, *0-P-45*, *0-P-90*, *45-P-45*, and *90-P-90* sandwich sheet rolling direction combinations.

The maximum limit strain is observed in the 0-P-0 sandwich sheet as 0.06 because the strain hardening value of the 0-P-0 sandwich sheet is 0.39 which is relatively higher than that of the other sandwich sheets except 90-P-90. The impact yield strength (*σ*), strength coefficient (*K*), and strain hardening exponent (*n*) along with plastic anisotropy (*R*) influence the deformation phenomenon in the sandwich sheets. These properties irrespective of parent materials are prompting the best formability for materials. In comparison with all these FLDs, the best formability is depicted in the 90-P-90 sandwich sheet, followed by the 0-P-0, 0-P-90, 45-P-45, and 0-P-45 sandwich sheets, respectively. This research can lead the industry sector to use the sandwich sheets with a combination of different rolling directions for superior formability.

The variation or improvement in the formability in terms of percentage is tabulated in Table 6 with limit strain comparison.

## 4. Conclusions

The present work addressed the formability analysis of AA5052 alloy sheets of 1 mm thickness and AA5052-PVC-AA5052 sandwich sheets of 2.5 mm thickness by considering rolling direction as a parameter. From the obtained results of this work, the importance of plastic anisotropy, i.e., rolling directions (0, 45, and 90) effect on formability analysis to base materials and sandwich sheets, is seen clearly. This work has brought the conclusion that 0-degree and 90-degree rolling have more or less similar forming behaviour in AA5052 alloy sheets, whereas 90-degree combinations dominated in sandwich sheets. From the mechanical properties such as yield strength (*YS*) and strain hardening exponent (*n*), one can estimate the sheet metal formability prior to the formability test. The formability of any sheet metal will not depend on a single parameter; however, it includes material properties, manufacturing process, thinning behaviour, strain rate, and formability parameters in a synergetic manner [26, 27].

## Figures and Tables

**Table 1:** List of related literature studies and their important outputs.

Reference	Type of sheet and method of examination	Parameters considered	Results obtained
Raghavan [1]	Marciniak biaxial stretch test and produced the in-plane FLCs by using a single punch/die on low-carbon drawing quality sheet steel (0.76 to 1.5 mm thickness)	Obtained in-plane FLCs and compared with the conventional out-of-plane dome tests	Results confirmed that (a) sheet thickness has an intrinsic influence on forming limits and (b) plastic anisotropy and (c) in-plane FLCs were somewhat lesser than out-of-plane FLCs near plane strain

Levy and Van Tyne [2]	Stress-based FLCs were developed using the uniaxial tensile test data of IF RePhos, HSLA 350, HS440W, DP 500, DP600, DP800, DP980, and TRIP600 with 0.7, 1.4, and 2.1 mm	Keeler–Brazier equation for developing the FLCs	FLCs were well correlated with strain-based FLCs

Pundan et al. (2017)	FLDs developed by Nakajima testing on DP600GA (1.2 mm thickness), DQ (0.7 mm thickness), HIF (0.8 mm thickness), and EDD (0.8 mm thickness)	Proposed a method based on a combination of experiments and the use of CrachLab	The forming limit diagram generated using the proposed methodology was compared with the one obtained using the standard procedure, and a good correlation was obtained

Lumelskyj et al. [3]	Evaluated the FLCs using finite element simulations with Nakazima formability tests on a DC04 steel sheet of 1 mm thick	Nakazima formability tests through experiments and simulations	The FLCs strains were calculated by using the strain localization criteria

Paul [4]	Reviewed and indicated the various parameters such as limit strain evaluation method, punch profile, microstructure, prestraining path, strain rate, and temperature influencing the FLCs	The tensile properties and their relationship with FLCs. Demonstrated the microstructure characteristics of FLCs	Sandwich sheets made of metal-polymer-metal formability evaluation and construction of FLD have been discussed

Sokolova et al. [5]	Investigated the deep drawing and bending of 316L/polyolefin/316L sandwich with 0.5 mm thick cover and 0.6 mm thick core	The geometry and the size of the local inlays	Observed less formability of solid steel inlays MPM sandwich composites than without reinforced sheets

Carradò et al. [6]	Stainless steel (0.5 mm thick) and/or aluminum alloy (AlMg_3_) (0.5 mm thick) and core polyolefin sheet (PP-PE). Tested MPM through adhesive and Erichsen test and deep drawing	For proper bonding in sandwich sheets, roll bonding and heating press processes are used. Used two flat punches of various sizes and shapes	The properties of the sandwiches have been studied and compared with similar, industrially produced materials available on the market

Sokolova et al. [7]	The formability of sandwich composites made of 316L/PP-PE/316L with various sample sizes and core thicknesses for the deep drawing process	Different sample sizes and core thickness	The punch geometry and the core thickness were found a significant impact on sandwich-forming behaviour
		Two flat punches of different sizes and shapes	

Liu et al. [8]	A numerical simulation model was created and simulated the forming of AA5052-polyethylene-AA5052 sandwich sheets with the conditions at the interface between the skin sheet and the core materials	Sandwich sheets were tested using the rigid punch and Nakazima forming methods under three different separation, adhesion, and stick interface conditions	Found that as interfacial adhesion strength increases, the FLD of sandwich sheets shifts to a higher value

Liu et al. [9]	The FLDs of AA5052-polyethylene-AA5052 sandwich sheets	The Gurson–Tvergaard–Needleman damage model was used for simulation. Nakazima forming tests	The results showed that the formability of the AA5052/polyethylene/AA5052 sandwich sheet was superior to that of the monolithic AA5052 sheet. Formability was increased by increasing the thickness of polyethylene core layer

Li et al. [10]	The face sheets and core of the honeycomb sandwich panels were made of Al-1200 and Al-5052	The blast resistance of square sandwich panels with hexagon aluminum honeycomb cores through experimentations and numerical simulations	They have studied the deformation phenomenon of aluminum honeycomb cores

Harhashe et al. [11]	Developed 316L/polymer/316L (SPS) sandwich materials by roll-bonding method with variable core thickness (0.3, 0.6, and 1.8 mm) with 0.5 mm thick 316L	Examined mechanical properties by changing core thickness. Investigated under deep drawing conditions	According to the deformation analysis, the inserted reinforcements had a significant impact on the strain distribution, failure, and consequently the location of cracking

Harhash et al. [12]	Developed deep drawing steel grade 316L with a skin sheet thickness of 0.49 and 0.24 mm, while the core sheet was a PP-PE copolymer foil of 0.3, 0.6, 1.0, and 2.0 mm thick	Examined the impact of various core thicknesses on the mechanical properties in terms of elastic modulus, yield, and ultimate tensile strength	Deep drawing was used to determine FLC curve of SPS

Harhash et al. [13]	Developed deep drawing steel grade 316L with a skin sheet thickness of 0.49 and 0.24 mm, while the core sheet was a PP-PE copolymer foil of 0.3, 0.6, 1.0, and 2.0 mm thick	The impact of reinforcements with various geometries, sizes, materials, and locations on the stretching and deep drawability of SPS	The stretching outcomes demonstrated that a smaller reinforcement decreases the forming potential

Harhash et al. [14]	Developed deep drawing steel grade 316L with a skin sheet thickness of 0.49 and 0.24 mm, while the core sheet was a PP-PE copolymer foil of 0.3, 0.6, 1.0, and 2.0 mm thick	The deep drawing behaviour of SPS sandwich sheets by experimental, analytical, and numerical methods	A good agreement was derived regarding predicting the forming forces, the strain field distribution, and thickness reduction results

Kami et al. [15]	Analyzed the formability of three-layer DC06 skins with polymer in the middle sheets	An anisotropic GTN model and a modified M-K model were used to quantify the FLCs of sandwich sheets	The results showed that all of the parameters had significant effects on the formability of the sandwich sheets. Furthermore, it was found that, by increasing the thickness of the layers, the sandwich sheets formability improved

Miranda et al. [16]	Fabricated steel metal skins with 0.3 mm thickness and a polymeric core with 1 mm thickness	Characterization and formability of SPS sandwich materials, hole expansion tests, and deep drawing Erichsen test	Formability was observed through numerical simulations that were also performed to know the influence of tool geometry during the hole expansion test

Forcellese and Simoncini [17]	The three-layer MPM sandwich composite obtained by cold rolling bonding a core film in polypropylene polyethylene	Punch tests with a hemispherical shape were used to gauge formability	Results were related to the debonding mechanism occurring at the interfaces between steel sheet and plastic core as the angle of the sample axis was 0° and 90°
	Resin, 0.4 mm in thickness, with two cover sheets in higher strength interstitial free steel (0.2 mm thick)		Compared samples oriented at 0° and 90° to the rolling direction, and it was found that samples oriented at 45° to the rolling direction had the highest mechanical properties and formability

By Marques et al. [18]	Finite element analysis was used and studied the forming behaviour of multilayer sheets on two multilayer sheets of interstitial free steel of a thickness of 1.6 mm, A1060-O of 0.3 mm thick, and a polymeric core, 1.0 mm thick	The behaviour of the multilayer sheets and their equivalent materials was assessed using numerical simulations of the bulge test, deep drawing of a U-channel profile, and square cup	The curves of force vs. displacement of the punch as well as the strain and stress distributions were used to evaluate the effects of the different mechanical properties of the constituent materials and some geometric parameters of the deep-drawing process on the plastic behaviour

Harhash et al. [19]	Investigated the forming behaviour of sandwich composites made of SPS. Developed deep drawing steel grade 316L with a skin sheet thickness of 0.49 and 0.24 mm, while the core sheet was a PP-PE copolymer foil of 0.3,0.6, 1.0, and 2.0 mm thick	Under three-point bending conditions, a wide range of SPS layer configurations and thicknesses were tested while taking into account various bending angles (60, 90, and 150°) and punch radii (1.5, 3, 6, and 12 mm)	The results were validated, and a good fit between the numerical and analytical findings and the experimental findings was made

Kazemi et al. [20]	Made AA5754-polyethylene-AA5754 sandwich composite sheets, respectively (0.5 mm each skin layer and 0.5 mm polyethylene layer), and developed the forming limit diagram through experimentally and numerically	Evaluated mechanical properties, Nakazima test, and fractography	The forming results illustrated that the AA5754-polyethylene-AA5754 sandwich composites are applicable to be used instead of the aluminum sheets

Bekele et al. [21]	Investigated formability analysis of metal-polymer sandwich composites made of “AW 6082-PVC-AW 6082 (APA)” and “galvanized steel-PVC-galvanized steel (GPG)” sandwich sheets	For evaluating the formability, the actual limit dome height (LDH)—biaxial strain path—tests were simulated	The results were analyzed by forming limit diagram, punch force distribution, and a dome height at diverse conditions of punch velocity and friction. A comparison was made and represented the best combinations for the formability of the sandwich composites

Kella and Mallick [22]	AA5182-O/polypropylene/AA5182-O laminates with various combinations of core and skin thicknesses	Studied the springback of the sandwich laminates, the effects of various tool design and process parameters, such as die radius, punch radius, and blank holder force	The springback response of sandwich laminates is higher than that of the single aluminum sheet if they have the same thickness

Pazand et al. [23]	The forming boundaries and deformation characteristics of three-layer metallic (aluminum (AA3004), stainless steel (SUS304), and copper (Cu1011)) sheets were attempted	A numerical finite element method was used to investigate how the layer arrangement affected the FLD, stress triaxiality, and limiting dome height (LDH)	The results showed that the material properties, particularly the tensile strength, play a key role in controlling the FLD of the three-layer sheets

**Table 2:** Mechanical properties of the base metal AA5052 sheet in different rolling directions.

Orientation of sheet	YS (MPa)	*n*
0°	181.36	0.24
45°	172.54	0.22
90°	185.96	0.24

**Table 3:** Mechanical properties of AA5052-polymer-AA5052 sandwich sheets.

Sandwich sheets	YS (MPa)	*n*
0°-P-0°	129.25	0.39
45°-P-45°	125.3	0.29
90°-P-90°	129.15	0.44
0°-P-45°	120.165	0.30
0°-P-90°	136.35	0.28

**Table 4:** Limit strain value of the AA5052 base metal.

Sheet orientation	FLC_0_
0°	0.043
45°	0.038
90°	0.043

**Table 5:** Limit strain value of AA5052-PVC-AA5052 sandwich sheets.

Sandwich sheets	Limit strain
0°-P-0°	0.06
45°-P-45°	0.050
90°-P-90°	0.058
0°-P-45°	0.057
0°-P-90°	0.052

**Table 6:** Percent variation of limit strains in base sheet and sandwich sheets.

Base sheet	Limit strain	Sandwich sheet	Limit strain	Variation (%)
0-degree	0.43	0-P-0	0.060	28.33
45-degree	0.38	45-P-45	0.050	24.0
90-degree	0.43	90-P-90	0.058	25.86

## Data Availability

The datasets used and/or analyzed during the current study are available from the corresponding author upon reasonable request.
